# PCR‐based prevalence of a fatal reovirus of the blue crab, *Callinectes sapidus* (Rathbun) along the northern Atlantic coast of the USA

**DOI:** 10.1111/jfd.12403

**Published:** 2015-08-06

**Authors:** E M Flowers, K Simmonds, G A Messick, L Sullivan, E J Schott

**Affiliations:** ^1^ Institute of Marine and Environmental TechnologyUniversity of Maryland Center for Environmental ScienceBaltimoreMDUSA; ^2^ Cooperative Oxford LaboratoryUSDOC/NOAA/NOS/NCCOSOxfordMDUSA; ^3^ Baltimore Polytechnic Institute High SchoolBaltimoreMDUSA

**Keywords:** crustacean, CsRV1, disease, RLV, mortality

## Abstract

There is a need for more information on the relationship between diseases and fluctuations of wild populations of marine animals. In the case of *Callinectes sapidus* reovirus 1 (CsRV1, also known as RLV), there is a lack of baseline information on range, prevalence and outbreaks, from which to develop an understanding of population‐level impacts. An RT‐qPCR assay was developed that is capable of detecting 10 copies of the CsRV1 genome. In collaboration with state, federal and academic partners, blue crabs were collected from sites throughout the north‐eastern United States to assess the northern range of this pathogen. In addition, archived crab samples from the Chesapeake Bay were assessed for CsRV1 by RT‐qPCR and histology. PCR‐based assessments indicate that CsRV1 was present at all but one site. Prevalence of CsRV1 as assessed by RT‐qPCR was highly variable between locations, and CsRV1 prevalence varied between years at a given location. Mean CsRV1 prevalence as assessed by RT‐qPCR was >15% each year, and peak prevalence was 79%. The wide geographic range and highly variable prevalence of CsRV1 indicate that more study is needed to understand CsRV1 dynamics and the role the virus plays in blue crab natural mortality.

## Introduction

Marine animals are affected by an array of infectious agents that may significantly change their abundance (Lafferty, Porter & Ford 2004). In sessile organisms such as corals and oysters, the effects of disease on populations are well documented (e.g. Gardner *et al*. 2003; Powell *et al*. 2008). However, it may be difficult to detect disease‐related morbidity and mortality in motile marine species because disease signs may not be apparent and dead animals are not readily observable in the depths. Populations of blue crabs (*Callinectes sapidus*) exhibit interannual fluctuations in abundance in their North American range, as exemplified by the well‐studied population in the Chesapeake Bay (Colton *et al*. 2013; MD DNR 2014). Much of this variation may reflect fluctuation in recruitment and predation (Orth, van Montfrans & Fishman 1999; Hines 2007; Facendola & Scharf 2012). However, now receiving recognition among resource managers, and proposed more than a decade ago, is the possibility that diseases play a significant role in blue crab natural mortality (Messick & Shields 2000; Shields 2003; CBSAC 2014).

Blue crabs harbour a variety of pathogens, including diverse protozoans and viruses (Messick & Sindermann 1992; Stentiford & Shields 2005; Shields & Overstreet 2007). Many blue crab viruses have been reported anecdotally or not in connection with disease (e.g. Jahromi 1977; Johnson & Lightner 1988). However, one virus, CsRV1 (also called RLV for Reo‐Like Virus, or CsRV [Tang *et al*. 2011]), has been repeatedly found in association with mortality of captive crabs from Maryland and Virginia coastal bays (Johnson 1977; Messick & Kennedy 1990) and was recently discovered to be present in a majority of crabs dying in soft shell crab production systems in Maryland and the Gulf coast of Florida (Bowers *et al*. 2010). The virus, which is fatal when injected into naïve crabs (Bowers *et al*. 2010), infects hemocytes, hemopoietic and connective tissues and causes pathology of tissues in association with hemocyte infiltration (Johnson 1977; Tang *et al*. 2011). The 55–60 nm icosahedral virions contain a double‐stranded RNA genome comprised of 12 segments. Partial genome sequence analysis indicates that CsRV1 is loosely related to the Cardoreoviruses (Mayo & Haenni 2006) and is highly similar to mud crab reovirus (MCRV or SsRV) which causes mass mortalities of cultured *Scylla serrata* in China (Bowers *et al*. 2010; Deng *et al*. 2012).

Similar to aquatic protozoan and bacterial pathogens, the distribution of viral pathogens in aquatic environments may be environmentally or geographically restricted by temperature (Parry & Dixon 1997; Choi *et al*. 2006; Ford & Chintala 2006; Goodwin & Merry 2011; Perrigault *et al*. 2011). CsRV1 has been documented from multiple locations in the mid‐Atlantic and Gulf of Mexico, which suggests that the virus may be present throughout the temperate and subtropical climate range of blue crab (Bowers *et al*. 2010). The US blue crab fishery has a mean value of $142 million per year, with the largest regional fisheries in the Chesapeake Bay and Louisiana, where CsRV1 has already been detected. However, states north of Maryland support a blue crab fishery worth $12.5 million per year (2004–2013 average, NOAA NMFS data), and it is not known if CsRV1 is present in these fisheries. Defining the northern geographic range of CsRV1 in blue crabs would answer the question of whether this virus extends throughout the northern range of blue crab. In the context of predicted climate change‐driven expansion of the blue crab geographic range and alterations in host–pathogen dynamics, it is also important to establish a reference point for future blue crab health studies (Van Guelpen, Pohle & Chmura 2006; Burge *et al*. 2014; Johnson 2015).

Although Bowers *et al*. (2010), using a method that directly visualized the double‐stranded RNA (dsRNA) genome of the virus, described CsRV1 at over 50% prevalence in dying captive soft crabs, they did not detect CsRV1 dsRNA in a small sample of healthy wild crabs. Messick (1998), using histological methods, detected evidence of CsRV1 in only 0.3% of overwintering crabs. It is unclear whether the high prevalence of CsRV1 observed in captive crabs reflects transmission in captivity or instances of high prevalence in wild populations. To assess CsRV1 in wild blue crabs, we developed a highly sensitive real‐time quantitative PCR assay (RT‐qPCR). This assay was then used to measure the prevalence of CsRV1 RNA and geographic range of CsRV1 in the north‐eastern United States, at sites from Maryland to Massachusetts.

## Materials and methods

### PCR primer design

Potential qPCR primer sets were designed based on the sequence of the tenth genome segment of CsRV1 (GenBank HM014011.1) with the assistance of a computational primer selection tool (www.idtdna.com). Primers tested had no hits, other than the target sequence, in a GenBank Primer BLAST search of the nr database (www.ncbi.nlm.nih.gov/tools/primer-blast/index.cgi). Primers were tested by end‐point RT‐PCR on CsRV1 viral dsRNA (prepared by CF‐11 chromatography as in Bowers *et al*. 2010). A primer pair that produced the predicted 158‐bp amplicon without secondary products was then tested for qPCR performance on the cloned DNA target. The primer pair chosen for optimization consisted of 5′‐TGCGTTGGATGCGAAGTGACAAAG‐3′ (RLVset1F) and 5′‐GCGCCATACCGAGCAAGTTCAAAT‐3′ (RLVset1R). Sequencing of the 158‐bp amplicon confirmed that the region amplified was the intended RLV target.

### Preparation of DNA and dsRNA standards

A plasmid‐borne copy of CsRV1 segment 10, designated Clone 20 (Bowers *et al*. 2010), was used as a standard for optimizing qPCR amplification conditions. Plasmid DNA was purified using a commercial kit (Zymo Plasmid MiniPrep Kit), then linearized with restriction enzyme EagI. Spectrophotometric quantification (A_260_, NanoDrop) was used to calculate copy number based on plasmid (pGEM‐T, 3000 nt) and insert size (1233 bp).

Purified CsRV1 genomic dsRNA was used to characterize assay efficiency and reproducibility and was used as the quantification standard for assessments of CsRV1 in crab RNA. To prepare the dsRNA standard, total RNA was extracted from muscle of a CsRV1‐infected crab preserved at −80 °C. dsRNA was enriched by CF11 column chromatography and ethanol precipitation (Bowers *et al*. 2010). Gel electrophoresis was used to confirm purity and integrity of the dsRNA genomic segments. Spectrophotometric quantification (A_260_, NanoDrop) of dsRNA was used to calculate genome copy number based on an estimated genome size of 23.7 Kb (Bowers *et al*. 2010).

For both the plasmid DNA and dsRNA standards, a log_10_ dilution series was made in nuclease‐free water ranging from 10 to 1 × 10^6^ copies μL^−1^. The dsRNA standards included 25 ng μL^−1^ carrier yeast tRNA in each concentration.

### RT‐qPCR assay for CsRV1

Quantitative PCR cycling conditions were optimized for efficiency and low background. The optimization of qPCR performance included an assessment of background fluorescence in negative controls and the presence of secondary products as indicated by anomalous melting temperatures. Prior to reverse transcription, a dsRNA melting and annealing step for template and primers was introduced to improve sensitivity and decrease background fluorescence.

The following standard RT‐qPCR, procedure was adopted for quantification of CsRV1 genome copy numbers in crab RNA. Samples and standards were run in duplicate; when duplicates produced data that did not agree, samples were re‐assayed in triplicate. The dsRNA standard or crab RNA was combined with an equal volume of primer mixture, which contained the forward and reverse primers each at 5 μm. RNA with primers was heated to 95 °C for 5 min and rapidly chilled to 4 °C. Two microlitres of the primer and template mixture was then used per 10 μL RT‐qPCR reaction. Each reaction had the final components: 1X TaqMan^®^ Fast Virus 1‐Step Master Mix, SYBR^®^ Green (Life Technologies S‐7563), diluted 1:50 000 from manufacturer stock and 500 nm of each primer. As explained below, each PCR reaction represents the RNA extracted from 1 mg of host tissue.

Reverse transcription and amplification were conducted on an Applied Biosystems^®^ 7500 Real‐Time PCR System with the following conditions: 5 min at 50 °C (reverse transcription) followed by 5 min at 95 °C (reverse transcriptase inactivation and template denaturation). Amplification was achieved by 40 cycles of 10 s at 95 °C (denaturation) followed by a 20 s at 61 °C (annealing and elongation). Following the final amplification cycle, PCR products were subjected to a melting temperature analysis, which was determined by plotting the negative first derivative of SYBR Green fluorescence as the temperature rises from 60 to 90 °C.

### Crab collections and environmental data

**Figure 1:**
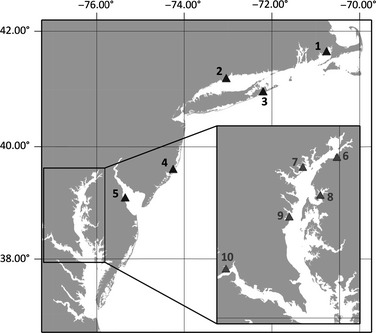
Sampling sites in the north‐eastern United States. Triangles 1–5 indicate sites sampled in 2011 and 2012. Inset shows sampling sites within the Chesapeake Bay where crabs were collected in 2010. Numbered locations in the north‐east are as follows: 1 Massachusetts; 2 Connecticut; 3 New York; 4 New Jersey; and 5 Delaware. The Chesapeake Bay sites are within the Maryland portion of the bay and are as follows: 6 Sassafras River; 7 Middle River; 8 Corsica River; 9 Rhode River; and 10 Nanjemoy River.

Crabs were collected by partnering organizations in late summer and fall of 2011 and 2012 (Fig. 1). Crabs were chilled on ice at the time of harvest, then frozen at −20 °C until shipment (frozen) to the Institute for Marine and Environmental Technology (IMET). At IMET, crabs were maintained at −20 °C until analysis. Institutional affiliation of partners (see Acknowledgements) was Massachusetts Division of Marine Fisheries (MA), Rider University (NJ), NOAA Milford Laboratory (CT) and Delaware Bay National Estuarine Research Reserve (DE). Crabs from NY were collected by one of the investigators (EJS). A variety of harvest methods were used, including baited traps (MA, NJ), dip nets (MA and NY) and trawls (CT and DE). Massachusetts sampling locations were in the tidal portion of the Agawam River (41.7519, −70.7058) and Westport River (41.5554, −71.0639); Connecticut samples were taken in Milford Harbour (41.2156, −73.0547); New York samples were taken from a Long Island coastal pond (40.9331, −72.2296); New Jersey samples were from Great Bay (39.5235, −74.4042); and Delaware samples were from the tidal portion of the St. Jones River (39.0702, −75.4054).

When feasible, water temperature and salinity data were collected at the time of harvest. In addition, environmental data were downloaded from nearby NOAA buoys as identified by the National Buoy Data Center (http://www.ndbc.noaa.gov). Data from the following buoys were accessed: MA‐NWPR1; CT‐BRHC3; NJ‐JCTN4; DE‐delslwq. Buoy data from the latter two sites are within the National Estuarine Research Reserve system and were obtained from http://cdmo.baruch.sc.edu/. Maximum and minimum temperature were recorded for the months during which sampling occurred. Statistical analyses used mean temperature from the same period. Due to the absence of salinity data from buoys, median salinity from data collected at the time of harvest was used to assess correlation. No buoy data were appropriate for the enclosed coastal pond site in NY, and temperature and salinity data were taken at the time of crab harvest.

Crabs were collected within the Chesapeake Bay tributaries by the NOAA Cooperative Oxford Laboratory as part of an ecosystem assessment in 2010 (Leight *et al*. 2014). Crabs were collected on a baited trot line, chilled in a cooler, transported to the laboratory and dissected within 3 h for histology. Frozen samples were archived, transported to IMET on dry ice and kept at −80 °C until RT‐qPCR analysis. Environmental data for 2010 crabs were collected at the time of harvest at four of the five sites (October 2010). For Nanjemoy River, water quality data were obtained from the MDDNR Eyes on the Bay program (http://mddnr.chesapeakebay.net/eyesonthebay/), buoy RET2.2 Maryland Point.

### Crab dissection, RNA extraction and quantification of CsRV1

Dissections were performed with single‐use autoclaved wooden instruments and razor blades. Before each dissection, the bench and the crab cuticle were cleaned with ELIMINase™. Approximately 50 mg of muscle and hypodermis was dissected from a walking leg and homogenized in 1.0 mL of TRIzol (LifeTechnologies), using a Savant FastPrep™ FP120 homogenizer. RNA extraction was performed per TRIzol manufacturer specification. Resulting RNA pellets were dissolved in 50 μL of nuclease‐free water and stored at −80 °C.

Quantification of CsRV1 genome in extracted crab RNA was accomplished by conducting the RT‐qPCR assay described above with 1 μL of RNA per reaction, which represented 1 mg of crab tissue. Amplification of crab RNA was compared to a dsRNA standard curve from 10 to 10^6^ copies. The potential for cross‐contamination of samples during the dissection and RNA extraction process was assessed by dissecting a CsRV1‐free hatchery‐produced crab both prior to and immediately following dissection of crabs known to contain >1 × 10^9^ copies of CsRV1 RNA per mg of tissue and then processing RNA as usual. Testing for contamination was conducted on two occasions by two researchers. In one instance, CsRV1 contamination equivalent to 2 × 10^2^ copies per mg was detected in the RNA from the CsRV1‐free crab that was processed following the highly infected animal. Consequently, a conservative threshold of 10^3^ copies per mg was used to assign which animals were CsRV1 positive. PCR analysis included melt curve analysis, and reactions with aberrant melting temperatures (more than 1 °C different from standards, see below) were not scored as CsRV1 positive.

### Histology

Crabs collected in 2010 from the Chesapeake Bay tributaries were dissected, with gill, gut, hepatopancreas, epidermis, heart, hemopoietic tissue, brain, thoracic ganglion and muscle tissues removed for histologic processing. Tissues were placed in 1G4F fixative for 18 h, embedded in paraffin, cut at 5 μm and stained with Mayer's haematoxylin and eosin (Luna 1968; Howard *et al*. 2004). Tissue slides were examined for pathology, presence of parasites or any abnormal histologic characteristics (Johnson 1980; Messick 1995). At the time of dissection, a leg was removed from each crab, frozen and archived at −80 °C for RT‐PCR analysis. Crab legs were transported to IMET on dry ice and kept at −80 °C until analysis.

### Statistical analysis

All statistical tests were conducted with R (R Core Team 2013). 95% confidence intervals were calculated for qPCR‐based prevalence with a Bonferroni correction using the prop.test function (Oehlert 2000). Chi‐square tests for differences in prevalence were calculated in the same manner, using prop.test and a Bonferroni correction for multiple comparisons between sites. Correlation between environmental parameters and CsRV1 prevalence was assessed by sample‐wide linear regression on all crabs.

## Results

### RT‐qPCR assay performance

As displayed in Table 1, when tested on a dilution series of CsRV1 genomic dsRNA, the SYBR Green‐based CsRV1 RT‐qPCR assay had an efficiency of 100.8% and consistently detected as few as 10 copies of the target. When tested with a dilution series of a plasmid carrying a cDNA copy of the target sequence, the assay displayed an efficiency of 96.4% and a sensitivity of 10 copies. Efficiency and sensitivity of the assay were also evaluated under typical use. For 10 RT‐qPCR standard curves conducted between July and September of 2013, the mean slope was −3.44 with a standard deviation of 0.11 and sensitivity of 10 copies. This is an average efficiency of 95.2% under typical use with the dsRNA standard. The melt curve analysis for PCR products produced from cloned and dsRNA targets consistently showed melting temperatures of 81.9–82.5 °C.

**Table 1:** RT‐qPCR efficiency with dsRNA and plasmid DNA standards. Comparison of efficiency for the standards run in triplicate. The threshold cycles for a log_10_ dilution series are used to assess efficiency relative to 100% theoretical efficiency for a slope of −3.32

Template	Slope	Y‐intercept	*R* ^2^	Efficiency (%)
Plasmid	−3.412	37.802	0.997	96.37
dsRNA	−3.302	34.472	0.999	100.84

### Prevalence of CsRV1 RNA in blue crabs in the North‐east

Prevalence of CsRV1 RNA was assessed in a total of 577 crabs from estuaries in five states in the north‐eastern United States (Fig. 1). CsRV1 RNA was found in crabs from all locations sampled (Tables 2 and 3). With the exception of Delaware Bay in 2012, CsRV1 RNA was identified in crabs from all states in both 2011 and 2012 samples. Crabs ranged in size from 8.5 mm to 175 mm in carapace width (CW, the distance between the lateral spines). CsRV1 PCR‐positive crabs were found across this size range, including the smallest and largest sizes. There was no difference in prevalence between male and females crabs (22% and 26%, respectively; *P* = 0.45). Because many crabs were collected by non‐random methods (trap, hand net), it was not meaningful to attempt to look for specific correlations between CsRV1 prevalence and crab size or sex.

**Table 2:** CsRV1 prevalence in the north‐eastern United States. Sampling site numbers correspond to locations labelled in Fig. 1. CsRV1 per cent prevalence is given with 95% confidence intervals (CI), as well as median and peak viral load for that sampling site. Salinity and temperature ranges were obtained from nearby NOAA buoys, with the exception of site 3 in New York where no buoy was appropriate and environmental data collected at the time of sampling are provided

Sample site		Year	Months	*N*	PCR % prevalence	95% CI	MedianViral load	PeakViral load	Salinity (psu)	Temperature (°C)
1	MA	2011	Aug	104	7.7	2.7–19.0	1.3 × 10^4^	2.2 × 10^8^	10–17	19.8–23.8
2	CT	2011	Sep	52	51.9	32.7–70.6	1.3 × 10^3^	7.7 × 10^8^	na	20.5–24.3
3	NY	2011	Aug	19	31.6	9.8–64.9	7.1 × 10^3^	2.9 × 10^8^	7	26
4	NJ	2011	Sep	38	2.6	0.1–23.2	1.6 × 10^3^	1.6 × 10^3^	26–30	17.8–25.7
5	DE	2011	Jun–Sep	78	9.0	3.0–23.0	2.7 × 10^3^	5.7 × 10^7^	2–24	17.0–31.1
		2011	Total	291	16.8	12.1–22.9				
1	MA	2012	July, Oct	51	25.5	11.8–46.0	2.7 × 10^3^	8.4 × 10^8^	na	13.6–24.9
2	CT	2012	Sep, Oct	32	15.6	4.2–41.6	8.4 × 10^3^	5.6 × 10^7^	na	15.3–26.3
3	NY	2012	Sep	37	21.6	7.9–46.0	1.2 × 10^7^	1.4 × 10^8^	11	28
4	NJ	2012	Oct	28	78.6	50.3–93.5	4.8 × 10^3^	1.9 × 10^8^	27–32	12.5–22.1
5	DE	2012	July	20	0.0	0.0–31.7	na	na	10–24	22.3–30.0
		2012	Total	168	28.6	20.8–37.8				

**Table 3:** CsRV1 prevalence at five Chesapeake Bay sites during October 2010. Sampling site numbers correspond to locations labelled in Fig. 1. For RT‐qPCR data, CsRV1 per cent prevalence is given with 95% confidence intervals, as well as median and peak viral load for that sampling site. Salinity and temperature ranges were recorded at the time of sampling, with the exception of site 10 in the Nanjemoy River, where MDDNR buoy data were used

Sample site		*N*	PCR % prevalence	95% CI	MedianViral load	PeakViral load	Histology% Prevalence	Salinity (psu)	Temperature (°C)
6	Sassafras Ri.	22	31.8	10.9–63.0	2.0 × 10^3^	2.7 × 10^7^	0	0.87	16.5
7	Middle Ri.	24	0.0	0.0–27.7	na	na	0	11.4	17.2
8	Corsica Ri.	24	25.0	7.7–55.6	1.5 × 10^4^	2.7 × 10^6^	0	10.5	23.2
9	Rhode Ri.	24	45.8	20.6–73.3	4.1 × 10^3^	1.3 × 10^9^	0	12.4	19.4
10	Nanjemoy Ri.	24	4.2	0.1–33.2	na	2.9 × 10^3^	0	7.6	18.0
	Total	118	20.3	13.3–31.9					

CsRV1 prevalence was variable over the geographic range sampled in both years. For 2011, prevalence ranged from 3% in New Jersey to 52% in Connecticut. In 2012, prevalence ranged from 0% in Delaware to 79% in New Jersey. Sites with high CsRV1 prevalence did not appear to be geographically grouped and were not consistent from year to year. New York and Delaware did not have significant differences between 2011 and 2012 (*P* > 0.05). The highest prevalence was recorded from New Jersey in 2012 (79%), but this was preceded by 3% prevalence at that site in 2011 (*P* < 0.0001). The sites in Massachusetts and New Jersey had significantly higher prevalence in 2012 than in 2011, while the site in Connecticut had higher prevalence in 2011 (*P* < 0.05).

Prevalence of CsRV1 in crab RNA was variable between years in aggregate. The mean prevalence was 15% in 2011 (*n* = 369) and was 29% in 2012 (*n* = 168). Mean prevalence was significantly higher in 2012 than in 2011 (*P* < 0.001). CsRV1 prevalence in crab RNA encompassing all sites for both years was 21%.

CsRV1 was detected in RNA of crabs from a variety of environmental conditions and did not have a temperature, salinity or geographic boundary in this data set. Salinity ranged from 2 to 32 gL^−1^, PCR‐positive crabs were identified across that salinity range, and no significant correlation with salinity was observed (*R* ^2^ = 0.12, *P* = 0.43). The highest prevalence (79% in New Jersey during 2012) was at a salinity of 27–32 gL^−1^. Temperatures ranged from 12.5 to 31.1 °C for the regions and months sampled. The highest prevalence observed occurred at temperatures of 12.5–22.1 °C. However, high prevalence was not exclusively seen at lower temperatures, and the 52% prevalence observed in Connecticut during 2011 was found at 20.5–24.3 °C. As with salinity, there was no significant correlation between temperature and CsRV1 prevalence (*R* ^2^ = 0.25, *P* = 0.14).

### Prevalence of CsRV1 in blue crabs in the Chesapeake Bay

Archived blue crab samples from five tributaries of the Chesapeake Bay, MD sampled in October 2010, were evaluated for CsRV1 by RT‐qPCR and histology (Table 3). The mean CsRV1 prevalence for the 118 crabs as assessed by RT‐qPCR was 21%. Prevalence at individual sites ranged from 0% to 46%. On this smaller spatial scale, similar to comparisons between north‐eastern sites, there was no significant correlation identified between prevalence and temperature (*R* ^2^ = 0.07, *P* = 0.66) or salinity (*R* ^2^ = 0.01, *P* = 0.85). These same crabs from the Chesapeake Bay tributaries were also evaluated by histology using standard haematoxylin and eosin staining. None of the crabs had identifiable CsRV1 infections (cytoplasmic inclusions) by histology.

### CsRV1 genome copy numbers

The number of CsRV1 RNA target copies detected in each infected crab was highly variable, ranging from just above the threshold minimum of 10^3^ copies mg^−1^ to over 10^9^ copies mg^−1^ host tissue. For sites with more than three crabs positive for CsRV1, the mean CsRV1 RNA copy number ranged from 1.8 × 10^5^ (Connecticut in 2011) to 1.3 × 10^8^ (Rhode River in 2010), and the median at these sites was 1.3 × 10^3^ and 4.1 × 10^3^, respectively. The median CsRV1 genome ‘load’ for all CsRV1 positive crabs was 4.1 × 10^3^.

## Discussion

A sensitive and quantitative RT‐qPCR method was developed to detect RNA of CsRV1, a virus first described as pathogenic to the blue crab nearly four decades ago and recognized to be associated with mortality in soft shell crabs (Johnson 1977; Bowers *et al*. 2010). The correspondence in sensitivity and efficiency between standard curves produced from dsRNA and DNA indicates that the reverse transcription step of the RT‐qPCR process was very efficient. The ability to rapidly and sensitively assess as few as 10 copies of the CsRV1 target in frozen archived tissue enabled the analysis of CsRV1 from blue crabs collected throughout the north‐east and the Chesapeake Bay by a coalition of academic, state and federal collaborators.

Prevalence of CsRV1 RNA was found to be highly variable between locations within years and highly variable at most locations from year to year. RNA of CsRV1 was detected in crabs at all locations sampled, indicating that the virus is present in coastal systems from Maryland to Massachusetts. This study is the first to show that CsRV1 can be detected in sites over a broad area of the blue crab's northern range. Virus RNA was found in crabs of both sexes and of all sizes, and there was no apparent correlation of CsRV1 prevalence with salinity, temperature or location. This is in contrast to another major infectious agent of blue crab, the protozoan *Hematodinium perezi*, which is restricted to high salinity and has not been detected north of New Jersey (Messick & Shields 2000; Stentiford & Shields 2005; Pagenkopp Lohan *et al*. 2013; EJ Schott and A Hanif unpublished). The overall CsRV1 prevalence was 20%, which is substantially higher than previous indications based on histological assays (Johnson 1977; Messick 1998). The highest prevalence of CsRV1 was 79%, and in only two of the 15 samples was no CsRV1 RNA detected.

Previous studies of CsRV1 in blue crabs were based on histology using light or electron microscopic investigations of captive crabs (Johnson 1977) or wild crabs (Messick 1998). In the current study, no histological evidence of infection was apparent in a subset of 26 crabs that were positive by RT‐qPCR for the CsRV1 genome. It is remarkable that a crab carrying an CsRV1 viral load corresponding to 1.3 × 10^9^ targets per mg tissue showed no histopathological sign of virus infection. This difference between the prevalence of CsRV1 RNA and the prevalence of CsRV1 as indicated by histological changes is possibly an indication that CsRV1 infections do not produce histopathology or do so only in very advanced infections. The greater sensitivity of PCR relative to histology for the detection of crustacean viruses has been reported in a number of host/virus systems (e.g. Nunan, Poulos & Lightner 1998; La Fauce, Layton & Owens 2007; OIE 2014).

The quantitative aspect of the RT‐qPCR assay for the CsRV1 genome is a useful feature that permits the estimation of viral load. Stable cell cultures are not available to enumerate viruses in crustaceans, although the development of primary hemocyte cultures may make this feasible (Walton & Smith 1999; Li & Shields 2007; Shashikumar & Desai 2013). Therefore, although it is difficult to know how many of the CsRV1 genomes detected represent infectious viruses, the RT‐qPCR method nonetheless provides a standardized metric for comparing virus load in individuals over the course of infection and in populations over the course of outbreaks. It opens the door to experimental studies on the relationship of virus load to longevity and to studies on epidemiology of this virus. In this study, the median viral genome copy number in each sample of crabs was below 10^4^ virus genomes per mg of muscle, and yet peak CsRV1 target copy number sometimes reached more than 10^8^ viruses per mg of muscle. This could be an indication that crabs can carry a low‐level infection for an extended time and that when virus levels exceed some threshold, soon die. Our preliminary research on the longevity in captivity of wild crabs captured with different starting viral loads also supports this interpretation (EJ Schott and O Zmora, unpublished). Additional experimental and field research is needed to determine what CsRV1 genome load induces morphological changes to infected host cells and leads to host mortality. This would be helpful in assessing the usefulness of this highly sensitive genomic detection method for predicting the impact of CsRV1 on valuable fishery populations.

Highly variable CsRV1 prevalence over long distances (hundreds of kilometre, between states) as well as shorter distances (tens of kilometre, within the Chesapeake Bay) raises many questions. Among these is whether the patchiness in prevalence would be observed on even smaller geographic scales (1–2 km). Another major question is the scope and duration of CsRV1 outbreaks. Based on the rapidity with which CsRV1 kills crabs in a laboratory setting [3–4 weeks according to Bowers *et al*. (2010)], and given that juvenile crabs forage within limited geographic ranges (Hines *et al*. 1995; Hines 2007), a feasible hypothesis is that a subpopulation of infected crabs can maintain low‐intensity infections that, upon some stress or trigger, may rapidly intensify and result in death of the local subpopulation of crabs. Alternatively, it cannot be ruled out that crabs can clear themselves of virus infections and recover. To address this hypothesis and better understand CsRV1 outbreak dynamics, the RT‐qPCR assay will need to be applied to a study designed to assess CsRV1 prevalence over shorter time spans and smaller geographic distances.

The apparent ubiquity of CsRV1 and the observation of frequent high prevalence suggests that CsRV1 has the potential to measurably impact blue crab abundance. Natural mortality estimates for the blue crab vary considerably, and the contribution of diseases, including CsRV1, to natural mortality and population fluctuation is unknown (Miller *et al*. 2005; Hewitt *et al*. 2007). With a more complete understanding of the dynamics of CsRV1 infections in individual wild crabs, it may ultimately be possible to translate CsRV1 prevalence and variability into impacts on the blue crab population and associated fishery. This potential is especially apparent in the Chesapeake Bay, where blue crab population fluctuation has been extensively studied (Miller *et al*. 2011, MD DNR 2014). In the Chesapeake Bay, a better understanding of disease‐related mortality is a recently recognized management priority (CBSAC 2014). The high prevalence of CsRV1 identified in this study and the high variability over space and time indicate that this virus has the potential for substantial impact on local or regional populations of blue crab.
